# Pemphigus vulgaris and focal segmental glomerulosclerosis (FSGS): First reported case and a review of the literature

**DOI:** 10.1002/ccr3.7716

**Published:** 2023-07-18

**Authors:** Afsaneh Sadeghzadeh‐Bazargan, Atefeh Amouzegar, Maryam Abolhasani, Abbas Dehghani, Azadeh Goodarzi, Seyyedeh Tahereh Rahimi

**Affiliations:** ^1^ Department of Dermatology, Rasool Akram Medical Complex Clinical Research Development Center (RCRDC), School of Medicine Iran University of Medical Sciences (IUMS) Tehran Iran; ^2^ Firoozgar Clinical Research Development Center, School of Medicine Iran University of Medical Sciences (IUMS) Tehran Iran; ^3^ Oncopathology Research Center, School of Medicine Iran University of Medical Sciences (IUMS) Tehran Iran; ^4^ Department of Pathology, Hasheminejad Kidney Center, School of Medicine Iran University of Medical Sciences (IUMS) Tehran Iran

**Keywords:** case report, focal segmental glomerulosclerosis, FSGS, immunobullous disease, MCD, minimal change disease, nephropathy, nephrotic syndrome, pemphigus vulgaris, PV, renal disease, review

## Abstract

**Key Clinical Message:**

There may be a connection between pemphigus vulgaris and nephrotic syndrome, as evidenced by the occurrence of focal segmental glomerulosclerosis in our pemphigus vulgaris patient and reviewing relevant literature. Therefore, if a patient with pemphigus vulgaris presents with bilateral lower extremity edema or proteinuria detected during urinalysis, it could indicate involvement of the kidneys.

**Abstract:**

Pemphigus vulgaris is a type of autoimmune blistering disease characterized by the presence of IgG autoantibodies against desmogleins 3 and 1. It is important to evaluate potential autoimmune associations in patients with pemphigus vulgaris so that appropriate laboratory and physical examinations can be performed to monitor for any increased risk of other autoimmune disorders. This case report describes a 55‐year‐old woman who presented with oral and axillary erosions, which were diagnosed as pemphigus vulgaris based on skin histopathology and immunofluorescence. During follow‐up, the patient was found to have proteinuria, which led to referral to a nephrologist. The patient was diagnosed with nephrotic syndrome and minimal change disease after a biopsy. Despite treatment, the patient's proteinuria persisted and serum creatinine levels increased, leading to a second biopsy which confirmed the diagnosis of focal segmental glomerulosclerosis. This study reports on the first case of pemphigus vulgaris with focal segmental glomerulosclerosis and reviews the literature on the co‐occurrence of acquired immunobullous diseases and nephrotic syndrome of any kind.

## Graphical abstract

Normal glomerulus with a delicate basement membrane.

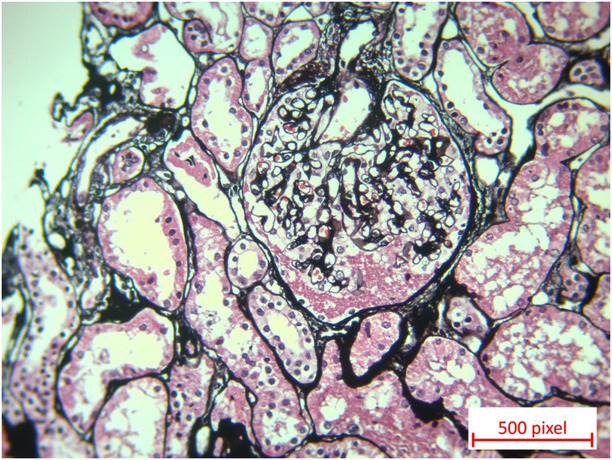

## 1 INTRODUCTION

Pemphigus vulgaris (PV) is a disease where the immune system produces antibodies that attack proteins in the skin, causing blistering.^1^ The main target of these antibodies is desmoglein 3, but up to 60% of patients may also have antibodies against desmoglein 1, which leads to pemphigus foliaceus (PF).^2^, ^3^, ^4^

PV patients may also be at risk for other autoimmune diseases, but it's unclear if this includes kidney problems.^5^ Minimal change disease (MCD) and focal segmental glomerulosclerosis (FSGS) are common causes of nephrotic syndrome with similar pathophysiology characterized by podocyte foot process extinction.^6^, ^7^ The exact pathogenesis of MCD and FSGS is unknown; one hypothesis is that certain circulating molecules injure podocyte foot processes and cause proteinuria and effacement.^6^, ^8^ Based on the therapeutic effect of drugs against B‐cell lymphocytes in some patients and the proteinuric effect of anti‐nephrin antibodies in rodent models, autoimmune pathogenesis with anti‐nephrin antibodies is hypothesized for these diseases.^9^

MCD is more common in children but can occur at any age.^10^ Some investigators believe that MCD and FSGS are two separate entities, whereas others consider them to be part of a continuum and MCD can transition into FSGS.^11^, ^12^ In this article, the authors describe a case of PV patient who developed MCD that later progressed to FSGS. We also review previous studies on the possible link between autoimmune skin diseases like PV and various types of kidney disease, including MCD, FSGS, membranous nephropathy, and IgA nephropathy.

## 2 CASE PRESENTATION

A 55‐year‐old woman presented to our dermatology clinic in May 2020 with a diagnosis of corticosteroid‐resistant PV. She had oral and axillary erosions but no other medical problems. Skin biopsy and immunofluorescence examination confirmed the diagnosis of PV, but after 6 months of treatment with topical treatments and systemic corticosteroids under the observation of another dermatologist, there was no clinical improvement.

Lab tests and dual‐energy X‐ray absorptiometry (DEXA) revealed proteinuria and osteoporosis. Due to the disease's corticosteroid‐resistant nature, the patient received 500 mg rituximab weekly for 4 weeks. Following this, we started the patient on mycophenolate mofetil at a dosage of 1 g per day and prednisolone at a dosage of 30 mg per day. After 3 months, the erosions improved and no new lesion developed. As a result, we discontinued mycophenolate mofetil and gradually tapered the patient's prednisolone dosage.

**FIGURE 1:**
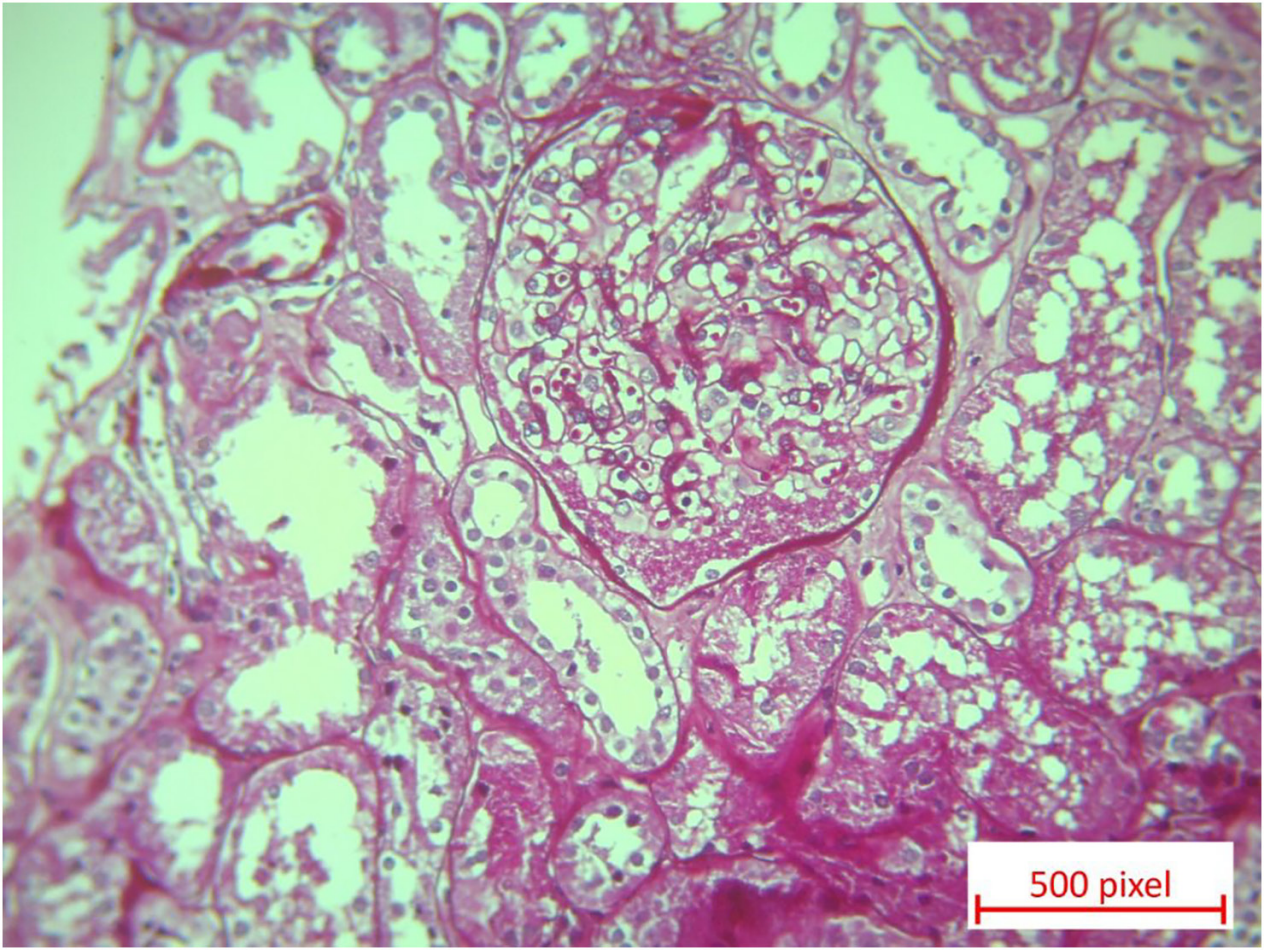
PAS staining shows one glomerulus with normal size and cellularity without mesangial expansion, surrounded by normal renal tubules (x20).

**FIGURE 2:**
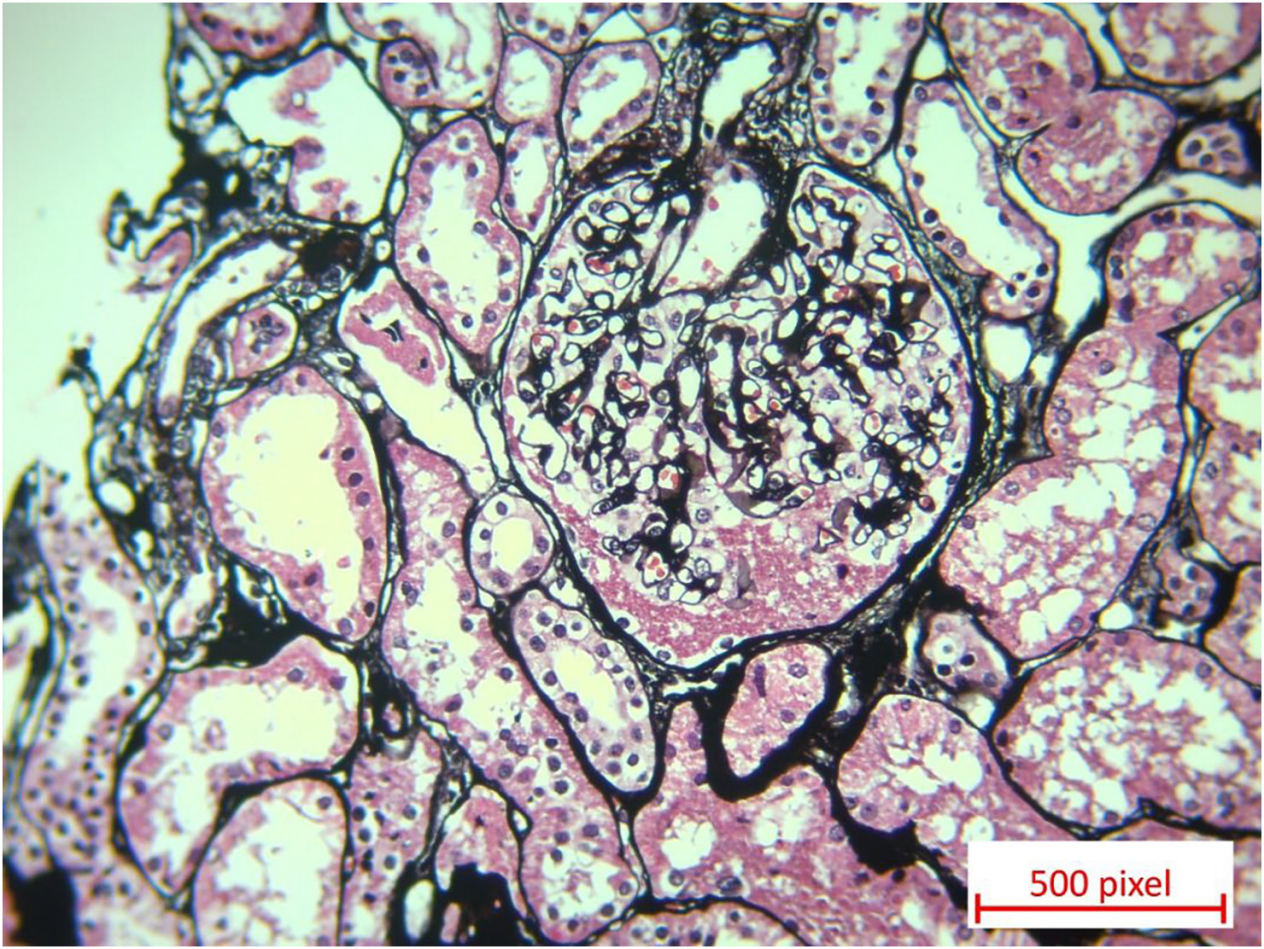
Jones' staining reveals one glomerulus without thickening of glomerular basement membrane, holes or sclerosis (x20).

However, during follow‐up, the patient developed proteinuria, which was diagnosed as nephrotic syndrome by a nephrologist and a kidney biopsy confirmed MCD shown in Figures 1 and 2.

**FIGURE 3:**
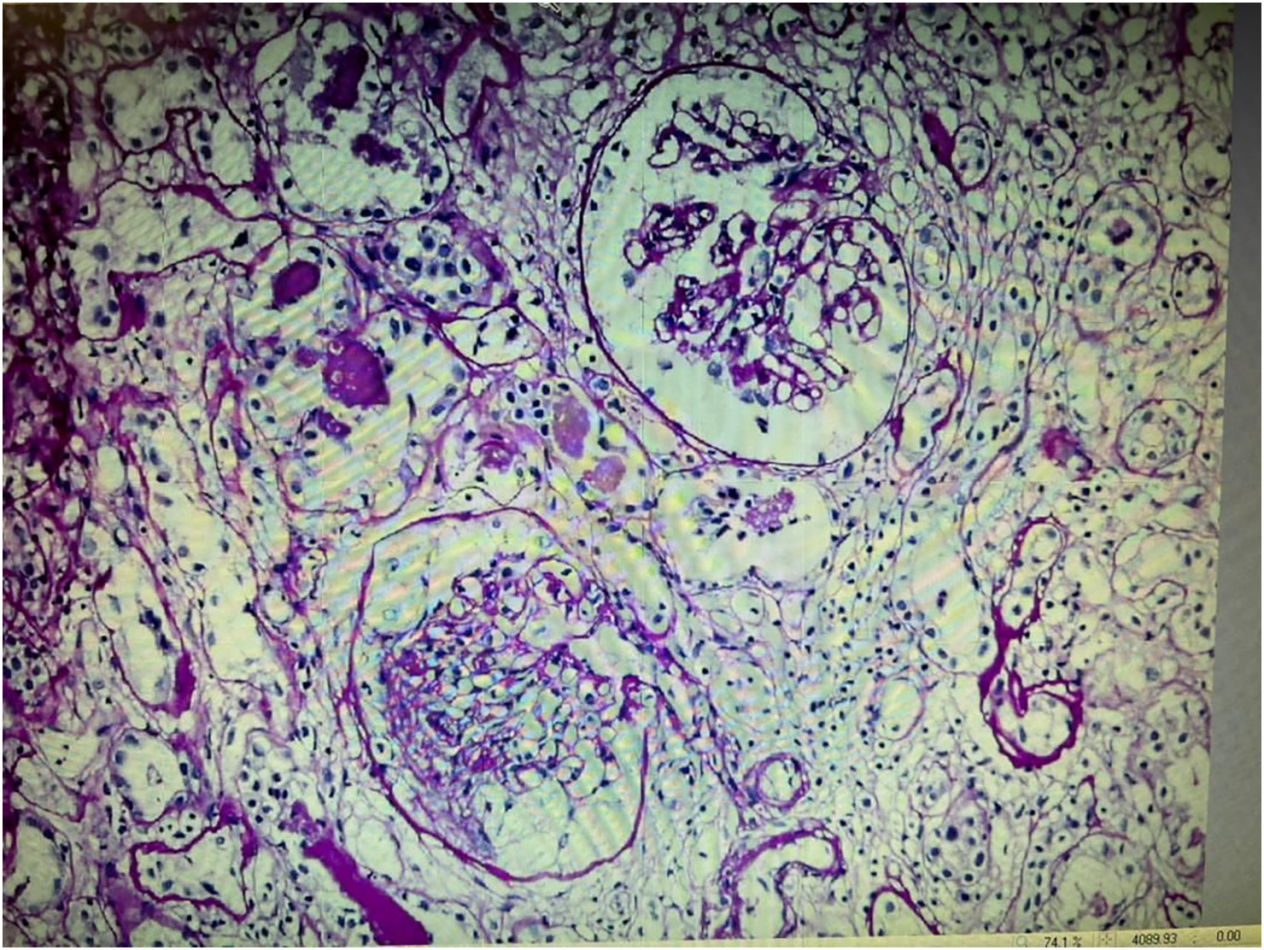
PAS staining (x20).

Despite 3 months of treatment with prednisolone 1 mg/kg/day, proteinuria persisted, and creatinine levels increased. Secondary investigations including anti‐myeloperoxidase and anti‐proteinase‐3 antibodies, antinuclear antibodies, anti‐double‐stranded DNA, and complements were normal. As a result, 1 year after the first biopsy, a second renal biopsy was carried out. Histopathological examination revealed interstitial fibrosis and tubular atrophy (IF /TA) of 40%, and no immune deposits were found. In addition, Congo red staining was negative, Figure 3. Based on the histopathologic findings, the patient was diagnosed with FSGS, type NOS (not otherwise specified). Finally, the patient was given a treatment of prednisolone and mycophenolate mofetil, but unfortunately, there was no clinical improvement. As a result, the patient was deemed eligible for a kidney transplant.

## 3 DISCUSSION

In this study, we have explored the potential association between pemphigus vulgaris PV and two commonly occurring causes of nephrotic syndromes, namely MCD and FSGS. Additionally, we have reviewed existing literature on the co‐occurrence of acquired immunobullous diseases and various types of nephrotic syndrome, including MCD, FSGS, membranous nephropathy, and IgA nephropathy, Table 1.

**TABLE 1:** Previous reported cases of co‐occurrence of acquired immunobullous diseases and any kind of nephrotic syndrome.

Case number	Author, year	Age	Gender	Immunobullous disease	Nephrotic syndrome	Renal disease presentation	Time of renal disease onset	Outcome
1	Esterly, N. B, 1973^19^	12	Female	Bullous pemphigoid	Membranous nephropathy	edema, hypertension, proteinuria, and hypercholesterolemia	One year before the skin disease	Death
2	Mark D. Herron, 2004^17^	37	Male	Pemphigus vulgaris	Minimal change disease	periorbital edema as well as edema of the lower extremities	2 months after the skin disease	NA
3	Misa Ikeda, 2017^20^	70	Male	Bullous pemphigoid	Membranous nephropathy	edema in the bilateral lower extremities	10 years after the skin disease	Oral prednisolone effectively treated the bullous pemphigoid and membranous nephropathy
4	Kawakami, T., 2009^21^	57	Female	Bullous pemphigoid	Membranous nephropathy	proteinuria	Simultaneous	Improvement by combined therapy with mizoribine (150 mg daily) and prednisolone (50 mg daily)
5	Mereniuk, E., 2021^22^	35	Female	Mucous membrane pemphigoid	Membranous nephropathy	generalized edema and proteinuria	NA	Successfully treated with rituximab
6	Oya, K., 2018^23^	38	Female	Bullous pemphigoid	IgA nephropathy	NA	2 years before the skin disease	Bullous pemphigoid treated with the combination of prednisolone 1 mg/kg/day, plasma exchange and IVIG
7	Perez, G. L., 1995^24^	38	Male	Pemphigus foliaceus	IgA nephropathy	proteinuria and hematuria	2 months before the skin disease	Treatment of skin disease with methotrexate, 2.5 mg orally per day, and topical triamcinolone acetonide
8	Ross, E. A, 1989^25^	69	Male	Bullous pemphigoid	Membranous nephropathy	proteinuria and elevated serum creatinine	3 years before the skin disease	Treatment of skin disease with systemic corticosteroid
9	Soine, E. J, 2009^26^	55	Male	Bullous pemphigoid	Membranous nephropathy	bilateral lower extremity edema	Simultaneous	Treatment of skin disease with methotrexate 15 mg weekly
10	Stump, M., 2019^27^	68	Male	Mucous membrane pemphigoid	Membranous nephropathy	non‐nephrotic proteinuria and hematuria	1.5 years after the skin disease	Treatment of skin and renal diseases with 3 cycles x 4 doses of 375 mg/m rituximab
11	Uchino, Y., 2004^28^	71	Female	Mucous membrane pemphigoid	NA	pitting edema on her legs	Simultaneous	Oral prednisolone at 40 mg/day, which resulted in improvement of both the mucosal lesions and the nephrotic syndrome.

Considering the co‐occurrence of PV with MCD and FSGS nephropathies, anti‐nephrin autoantibodies could be implicated in the pathogenesis of MCD and FSGS, with similarities to anti‐desmoglein antibodies in PV.^6^, ^10^ Desmogleins are a component of the desmosomal cell adhesion complex and play an important role in the cell–cell adhesion of keratinocytes.^1^ Similarly, nephrin is a component of the gap junction complex between podocytes and plays a role in gap junction integrity. These similarities in pathogenesis may account for the co‐occurrence of PV and FSGS.^13^, ^14^

As FSGS primarily affects the glomeruli located in the corticomedullary junction, the lesion may be overlooked or misdiagnosed as MCD if samples are obtained from superficial tissue.^7^ Furthermore, some researchers suggest that MCD represents the early stage of FSGS, indicating a possible transitional nature. Our case analysis suggests that MCD was likely transitioning into FSGS.^12^, ^15^

Some studies have shown the association between PV and nephropathy. In one study, a 51‐year‐old man developed PV, MCD, and acute tubular necrosis after 11 months of treatment with penicillamine for rheumatoid arthritis, which was associated with features of Reiter's syndrome.^16^ Another study involved a 37‐year‐old man presenting painful oral, nasal, and scalp erosions of six‐month duration, with desquamative gingivitis prominent alongside periorbital edema and lower extremities. Histopathology and immunofluorescence confirmed PV diagnosis, while a kidney biopsy specimen indicated MCD.^17^ A third study analyzed a 61‐year‐old man with mucocutaneous lesions of PV and nephrotic syndrome; histopathology and immunofluorescence confirmed glomerular and vascular amyloidosis AA.^18^

Despite the scarcity of studies, our findings suggest a possible association between PV, MCD, FSGS, and other causes of nephrotic syndromes with autoimmune diseases. Detection of bilateral lower extremity edema or proteinuria in PV patients may aid in early diagnosis of renal involvement.

## 4 CONCLUSION

Considering the occurrence of FSGS in our PV case and the other types of nephrotic syndrome in previous studies, there might be an association between PV and nephrotic syndrome. A review of the literature indicates that the finding of bilateral lower extremity edema or proteinuria in the urinalysis of a PV case could be related to renal involvement.

## AUTHOR CONTRIBUTIONS

**Afsaneh Sadeghzadeh‐Bazargan:** Conceptualization; data curation; supervision; writing – original draft; writing – review and editing. **Atefeh Amouzegar:** Conceptualization; writing – original draft; writing – review and editing. **Maryam Abolhasani:** Data curation; funding acquisition; investigation; writing – review and editing. **Abbas Dehghani:** Investigation; writing – review and editing. **Azadeh Goodarzi:** Conceptualization; data curation; supervision; writing – original draft; writing – review and editing. **Seyyedeh Tahereh Rahimi:** Investigation; writing – review and editing.

## FUNDING INFORMATION

None.

## CONFLICT OF INTEREST STATEMENT

Authors declare no conflict of interest.

## ETHICS STATEMENT

Due to research protocol in Iran University of medical sciences, ethical committee's approval for case reports is not needed however patient's consent for publication is obtained.

## CONSENT

Written informed consent was obtained from the patient to publish this report in accordance with the journal's patient consent policy.

## Data Availability

All data used during this study are included in this article. Further enquiries can be directed to the corresponding author
